# Policy and perspective on outpatient programs for autologous hematopoietic cell transplantation and immune-effector cell therapy administration

**DOI:** 10.3389/fimmu.2024.1423959

**Published:** 2024-08-06

**Authors:** Scott R. Goldsmith, May San-Rozano, Justine Katindoy, Janet Rattanapichetkul, Michael Rosenzweig

**Affiliations:** Department of Hematology and Hematopoietic Cell Transplantation, City of Hope Comprehensive Cancer Center, Duarte, CA, United States

**Keywords:** multiple myeloma, autologous stem cell transplantation, CAR T therapy, IEC therapy, outpatient

## Abstract

High-dose chemotherapy with autologous hematopoietic cell transplantation (AutoHCT) has long been an integral treatment modality for multiple myeloma and non-Hodgkin lymphoma. Over the past 25 years, numerous institutions have shifted this practice from requiring hospitalization to one that can be performed in an ambulatory setting, resulting in cost savings and improved quality of life for patients. The recent advent immune-effector cell (IEC) therapies and expansion of their indications is changing the treatment landscape for hematologic and non-hematologic malignancies. However, current financial models and reimbursement structures threaten the viability and sustainability of this treatment modality should it continue to require inpatient administration and management. This threat is leading institutions to develop outpatient IEC programs based off the outpatient AutoHCT templates. Integral to the success of both is a cohesive program with outpatient-specific standard operating protocols, highly-trained providers and staff with expertise specific in these treatment modalities, evidenced-based supportive care and prophylaxis plans, extensive caregiver vetting and education, and the infrastructure to support all individuals involved. In this policy and practice review we provide an overview of the guidelines and published academic experiences, give a perspective-based description of the roles and responsibilities of the individuals involved in this process at our institution, and highlight actionable recommendations that could allow for the dissemination and implementation of outpatient AutoHCT and IEC programs more broadly.

## Introduction

Due to numerous factors including convenience, resource utilization, and microorganism exposure, numerous hematopoietic cell transplantation (HCT) and immune-effector cell (IEC) therapy centers are shifting HCT and IEC administration to the outpatient setting. Several publications have corroborated the feasibility and quantified the benefits of outpatient HCT/IEC in terms of patient satisfaction and cost-effectiveness without compromise on morbidity and mortality (1–3). Integral to the success of such programs are networks of providers, nurses, coordinators, and others who ensure streamlined workup, approval, therapy administration, and management of complexities and complications. Much like a *brigade de cuisine*, individuals at all hierarchical levels specialize in specific, generally non-overlapping aspects as a patient progresses through the different stages of the HCT and IEC processes, with the treating physician overseeing the totality of the operation and the patient remaining at the center. Moreover, ongoing communication between outpatient and inpatient services and seamless patient transitions between the ambulatory and inpatient settings are key toward ensuring ongoing patient safety and continuity.

Within our institution we developed an outpatient program initially for autologous HCT (AutoHCT) for multiple myeloma (MM) given the homogeneity of the therapy and process. Ultimately, with nearly a decade of experience, this became a springboard for other initiatives including autologous HCT for non-Hodgkin lymphoma (NHL) and germ cell tumors, total body irradiation conditioning for allogeneic HCT, bispecific antibody/immunotherapy administration, and both commercial and investigational IEC administration (including lymphodepletion [LD] and subsequent management). Rigorous patient selection, detailed standardized operating procedures (SOPs), focused expertise of those involved, and continual review of successes and challenges have been the cornerstones of these programs and contributed to their exponential growth. In this policy and practice review, we review the studies, policies, and guidelines surrounding outpatient HCT and IEC, provide a granular perspective of the roles and responsibilities of the involved individuals, generate evidence-based and experiential actionable recommendations founded in our procedures, and highlight opportunities for refinement and expansion.

## Policy options and implications

### Guidelines and policies from academia

#### Autologous HCT for multiple myeloma and non-hodgkin lymphoma

Numerous institutions have published on the feasibility and success of outpatient autologous HCT for MM and NHL. They describe several models in which outpatient management of AutoHCT recipients is partially or totally incorporated, including an early discharge model, mixed inpatient-outpatient model, and a total outpatient model (TOM) (4). **Table 1** reviews several studies that have reported outcomes on TOMs, a model that is becoming more heavily adopted. While an international set of practice guidelines has yet to be generated, some regional guidelines and institutional practices have been published (9).

**Table 1:** Retrospective studies of total outpatient management (TOM) models for outpatient autologous hematopoietic cell transplantation (AutoHCT) for multiple myeloma (MM) and non-Hodgkin lymphoma (NHL), describing experience, methods, outcomes, and risk factor for hospitalization.

	Gertz et al., 2008 (5)	Lopez-Otero et al., 2009 (6)	Holbro et al., 2013 (7)	Shah et al., 2017	Cavalier et al., 2020	Larsen et al., 2022 (8)
Study design	Single-center retrospective feasibility	Single-center retrospective	Single-center retrospective/safety and cost-effectiveness	Single-center retrospective	Single-center retrospective	Single-center retrospective
N	716	26	91	377 OP vs. 669 IP	18	811 first AutoHCT, 354 second AutoHCT
Time period	2000-2007	1993-2008	2006-2010	2008-2012	2018-2019	2015-2019
Disease(s)	MM	MM	MM	MM	NHL	MM
Conditioning	HDM	HDM	HDM	HDM (95% OP vs 81% IP), and other combination conditioning	BEAM	HDM, BEAM, “hybrid regimen” (VTD-PACE + low dose Mel)
Eligibility	Dialysis-dependent renal failure and age up to 76 not absolute exclusions Consistent caregiver required Exclusions: Lower performance status unrelated to disease, uncontrolled CHF, uncontrolled infection, severe chronic pulmonary disease	Normal renal function and liver function tests required Amyloidosis permitted in the setting of concomitant MM NOTE	LVEF> 40%, DLCO_Cor_ ≥50% Age<66 (up to 69 if good health) Consistent caregiver Lodging within 45min Controlled comordbities	No defined algorithm for IP vs. OP	NA	LVEF> 40%, DLCO_Cor_ ≥50% LFTs <2xULN sCr ≤ 3mg/dl KPS≥80 Consistent caregiver Negative infectious screening Lodging within 45min
Antimicrobial prophylaxis	FQ, PCN, ACV, Fluconazole	Cipro and fluconazole until engraftment	Cipro and fluconazole from D0 VCV if HSV seropositive PJP after engraftment	NA	NA	Levofloxacin, ACV, fluconazole
Hospitalization	438 (61%)	4 (15%)	76 (84%) at median 8d post AutoHCT	207 (55%) of OP at median 8d post transplant	15 (83%)	158 (31.5%) of first AutoHCT; 69 (28%) of second AutoHCT at median 7.7d
Risk factors for hospitalization	Renal dysfunction at baseline, age>65	3 of 4 infectious, 1 cerebrovascular accident	No statistically significant risks	NA	NA	1^st^ AutoHCT: low albumin, female gender; higher age and BEAM increased risk but not to statistical significance; 2^nd^ AutoHCT: KPS<90, history of hospitalization during 1^st^ AutoHCT
Survival	99% at D100	90.4% at D100, 80% at 76mo	100% at D100	83% at 2y in OP	100% at median 62d	Overall survival not reported, NRM 0.6% at D100
Notes	Mortality higher in those with progressive disease and transplanted during earlier time period	Apheresis occurred days -3 to-1 and products stored in conventional blood bank refrigerator without cryopreservation. Engraftment time median 27d for neutrophils, 37d for platelets Calculated cost per HCT $15,000 USD	Per patient cost saving of $19,522 CAD	Average cost savings $123,582 USD per patient	Most patients discharged back to complete outpatient transplant course Hospital days and cost reduced	

OP, outpatient; IP, inpatient; HDM, high-dose melphalan; BEAM, carmustine-etoposide-cytarabine-melphalan; CHF, congestive heart failure; LVEF, left ventricular ejection fraction; NA, not available; DLCO_cor_, corrected diffusing capacity of the lungs for carbon monoxide; LFTs, liver function test; sCR, serum creatinine; KPS, Karnofsky performance status; FQ, fluoroquinolone, PCN, penicillin, ACV, acyclovir; VCV, valacyclovir; PJP, Pneumocystis jiroveci pneumonia; CAD, Canadian dollars; USD, United States Dollars.

The published experiences of several institutions highlight the importance of patient selection in successfully navigating the outpatient AutoHCT process (5, 7, 8). Considerations for eligibility for outpatient AutoHCT are highlighted in **Table 2**. In general, comorbidities such as congestive heart failure, severe chronic pulmonary disease, poor performance status and renal dysfunction were heavily considered in determining, not only whether a patient could undergo outpatient AutoHCT, but whether it was appropriate at all. Several studies identified renal dysfunction as a risk factor for subsequent hospitalization although baseline renal dysfunction, even dialysis-dependence, was not generally exclusionary from initiation of TOM protocols. These medical comorbidities and a nuanced approach to AutoHCT are becoming more relevant as the treatment landscape of MM and NHL evolves (10, 11). Advanced age, in and of itself, has been associated occasionally with risk of hospitalization and morbidity in the AutoHCT setting, although across institutions and studies only some have used age cutoffs for eligibility for outpatient AutoHCT (12).

**Table 2:** Considerations for eligibility for outpatient AutoHCT based on published regional guidelines and institutional Standard Operating Procedures.

Prerequisites
Well controlled hematologic malignancy (eg. Partial response or better)
Adequate performance status (ECOG ≤2, KPS≥60)
Committed, 24-hour caregiver
Lodging within 1 hour
Potential exclusions
Severe, uncontrolled comorbidities
Severe chronic pulmonary condition (eg. FEV1<50% DLCO_cor_<50%)
Cardiomyopathy (eg. LVEF <45%), history of cardiac arrhythmia, or high risk of development (i.e. cardiac amyloidosis)
Dialysis-dependent renal disease (some institutions exclude those with CKD that are not dialysis-dependent)
Considerations for exclusion
Colonization of multidrug resistant bacteria or fungi
Recent severe infection

Beyond the medical factors in patient selection, the availability of a competent and committed caregiver available to the patient 24 hours a day is a ubiquitous prerequisite for TOM (5, 13). The infrastructure to support the patient and caregiver was also imperative, with all studies highlighting the daily evaluation by a provider in a dedicated transplant clinic/ambulatory hospital as well as the availability of a 24-hour triage line for after-hours medical needs.

Over the 25-year period in which outpatient AutoHCT has been conducted and reported, advances in supportive care, in conjunction with patient selection, seemingly have contributed to lower rates of hospitalization, morbidity, and early mortality. Earlier studies reported hospitalization rates of 50-80%, whereas a more recent study by Larsen and colleagues reported that only about one-third of patients required hospitalization, whether receiving a first or second AutoHCT (8). The most frequent reasons for hospital admission included neutropenic fever, uncontrolled gastrointestinal toxicity/mucositis, decline in performance status, or unavailability of caregiver. To mitigate risks of these toxicities and increase success of outpatient AutoHCT, supportive care and prophylaxis considerations based on these prior studies and our institutional experience are provided in **Table 3**.

**Table 3:** Guidelines for prophylaxis, supportive care, and infrastructure to maximize the success of outpatient HCT and IEC programs.

Antimicrobial Prophylaxis
HSV/VZV prophylaxis – most provide for all AutoHCT recipients, some limit to seropositive patients
Antifungal prophylaxis – generally fluconazole from D0 until engraftment or first month post-transplantation
Antibacterial prophylaxis – generally directed against gram-negative bacilli with fluoroquinolone or cefdinir. While many initiate at D0 until engraftment, others initiate when ANC < 500/µL in order to promote stewardship
PJP prophylaxis – heterogeneously applied, generally once patient has successfully engrafted for varying durations
Growth factor support
Commonly daily filgrastim starting on D+5 or D+7 until engraftment
Long-acting/pegylated filgrastim has been utilized successfully, generally administered D+1
For CAR T/IEC, growth factor support should not be initiated until patients are at low risk for cytokine release syndrome
Supportive care/symptom prophylaxis and management
Anti-emetic prophylaxis for highly-emetogenic conditioning regimens – including NK1RA, 5-HT3RA, benzodiazepine
Anti-emetic treatments in advance including prochlorperazine, 5-HTRA, benzodiazepine, with potential addition of transdermal scopolamine or olanzapine
Mucositis prophylaxis with cryotherapy
Anti-diarrheal agents for treatment of diarrhea prescribed in advance including loperamide and diphenoxylate/atropine, potential addition of octreotide or opium tincture possible in the outpatient setting
Infrastructure
Ambulatory transplant clinic/day hospital
Dedicated, highly trained nursing, APP, and supportive staff
Nearby lodging for those from further locations
24-hour triage line
24-hour evaluation/treatment center with staff familiar with HCT, ability to admit patient rapidly

HSV, herpes simplex virus; VZV varicella zoster virus; ANC, absolute neutrophil count; PJP, Pneumocystis jiroveci pneumonia; NK1RA, neurokinin 1 receptor antagonist; 5-HT3RA, 5-hydroxytryptamine(3) receptor antagonist; APP, advanced practice provider.

#### IEC/CAR T therapy

Due to the rapid expansion of CAR T therapy utilization as well as its advancing indications as earlier-line therapy in MM and NHL, many institutions are shifting their CAR T/IEC programs toward outpatient models, limiting the inpatient care to toxicity management and for those patients who are not eligible for outpatient administration (14). This initiative stems from the challenges with existing reimbursement structures for product acquisition and the cost of caring for CAR T recipients, which are less favorable for inpatient administration models (15). Hansen and colleagues conducted a systematic review examining the impact of outpatient versus inpatient CAR T administration as it pertained to clinical, economical, and patient-related outcomes (16). Within this review, 38 publications from 21 studies were included, the majority published after 2022, and included experiences ranging from single-center retrospective reports to subgroup analyses of multicenter phase 3 clinical trials. Safety appeared at least comparable between those in the inpatient and outpatient settings. Among studies reporting both inpatient and outpatient outcomes, some suggested higher incidence of cytokine release syndrome (CRS) and hematologic toxicity among those in the inpatient setting, although these were not adjusted for baseline characteristics and disease burden which could influence the location of CAR T administration. The systematic review did not identify any differences in overall response rates and overall survival. Health-related quality of life improved among CAR T recipients whether inpatient or outpatient. Costs of administration and care were significantly lower among the various outpatient care models, with hospitalization costs driving the main differences.

The American Society of Transplantation and Cellular therapies has developed a Best Practices Guidelines for Outpatient Administration of CAR T therapy (17). They proposed step-wise workflow that begins with the initial medical assessment by cellular therapy physician. Many of the eligibility criteria outlined for AutoHCT in **Table 2** remain relevant for patients intended for CAR T in the outpatient setting. Thereafter, patients undergo psychosocial and financial workup and clearance including the potential need for single-case agreements with insurance, enrollment with the manufacturing pharmaceutical company, and product purchasing. Part of the psychosocial assessment includes the identification and education of the caregiver, an integral aspect of the outpatient CAR T model. The dedicated caregiver is responsible for continuous care from the initiation of lymphodepletion until at least 30 days after infusion, and patients must be within a 2-hour driving radius to the treatment center during this time period (although usually within a shorter distance until D+14). Additionally, the REMS requirements for many products restrict patients from driving for the first 8 weeks after receiving CAR T. Preventative and prophylactic strategies to maximize the success of outpatient CAR T mirror that of AutoHCT outlined in **Table 3**. Moreover, the unique toxicities of CAR T including CRS and neurotoxicity require ongoing vigilance including specific education to the caregiver and patient, who must also be provided a product-specific wallet card, as well as serial measurements of vitals and cognition. Many are employing prophylaxis strategies for those at high risk of CRS based on published data, including the use of prophylactic dexamethasone during the first three days following CAR T administration. Notably, some CAR T products have delayed median onset of these inflammatory toxicities which may be beneficial from a reimbursement standpoint via outpatient administration, although also require ongoing vigilance extending even beyond the initial week or two after infusion. CAR T/IEC products in clinical development are aimed at lowering the toxicity and improving accessibility to outpatient CAR T.

### Overview of patient journey in outpatient HCT or IEC

The institution and success of outpatient HCT and IEC programs hinges on the expertise and perspectives of the involved providers and staff, as well as the values and perspectives of the patients themselves. In addition to the above-referenced guidelines and policies, herein we describe the patient journey as well as the roles and responsibilities of providers within our institutions.

#### Autologous HCT

In our institution, the most common indication for outpatient autologous HCT (autoHCT) is multiple myeloma using high-dose melphalan for conditioning. More recently, outpatient autoHCT with BEAM or thiotepa/BCNU conditioning for NHL is being performed in carefully-selected patients.

Patients may be already established with the treating hematologist or referred specifically for HCT. Disease-specific HCT nurse coordinators coordinate the elements starting with obtaining insurance authorization, completing the diagnostic workup, mobilization and collection, up until admission to the outpatient program for HCT. Specific SOPs exist encompassing the workup of patients to determine appropriateness for HCT in general and more specifically HCT in the ambulatory outpatient setting, referred to hereafter as day hospital (DH). Absolute contraindications for outpatient HCT include those requiring hemodialysis and those with cardiac amyloidosis and significantly compromised function. Other identified medical issues either by history, physical, or diagnostics may prompt further workup, consultation, or inpatient administration. Patients with straightforward indications for autoHCT and SOP-defined adequate organ function and performance status may bypass committee discussions and proceed with HCT in the outpatient setting. Those with complex disease or comorbidities as well as those with organ-function metrics that deviate from approved standards are presented to disease-specific and/or high-risk HCT committees to ensure the correct treatment and environment are selected.

Integral to the initial HCT workup is a psychosocial evaluation, performed by a licensed social worker, which not only assesses the patient but also their caregiver situation. Patients are required to have a competent caregiver with them for the duration of their autoHCT, from high-dose therapy administration until engraftment, and who will also be able to remain with them continuously at least for the first 30 days post-HCT. The caregiver responsibility is important, especially while the patient is not within the confines of the medical institution; therefore, we employ standardized instruction with printed material and readback from the caregiver and patient to ensure their comprehension and ability to comply.

In the administration and conduct of outpatient HCT, lodging may be a more heterogenous component dependent on the unique geographical and institutional resources. Our center relies on a hotel in immediate proximity which has cancer center guidance from food and cleanliness standpoints, as well as overlapping leadership and direct patient transportation. This houses the majority of autoHCT and outpatient IEC recipients. Patients who live within a 60-minute driving radius may be allowed to commute daily from home; however, the unpredictability of traffic and inability to closely regulate a patient’s home environment requires even more careful patient selection and favors use of the hotel. Unlike others, we do not have the geographical ability or infrastructure to conduct “HCT at home”, although this remains an interesting concept for those serving more rural environments.

Patients are admitted to the DH starting on day -1 with administration of high-dose chemotherapy. They are seen daily by an Advanced Practice Provider (APP) with expertise in acute ambulatory management of HCT recipients. The rounding HCT physician sees and examines patients on key days including high-dose chemotherapy days, hematopoietic progenitor cell (HPC) infusion days, and several days thereafter to assess toxicity. Together the APP and physician guide supportive management with assistance from HCT registered nurses (RNs), also with dedicated training and experience in ambulatory HCT. Patients receive antimicrobial prophylaxis that is adapted to depth and duration of neutropenia and balances infectious risk with antibiotic stewardship (**Table 3**). Staff are trained to remain vigilant for infection and other toxicities, with a low threshold to initiate an inpatient admission should fever or other signs or symptoms arise requiring more intensive monitoring and care. Caregivers are instructed to routinely assess temperature when off premises and have streamlined access to a 24-hour triage hotline and physician-staffed evaluation and treatment center should issues arise after-hours. The RNs and providers involved in these after-hours resources have dedicated instruction and expertise in fielding questions from HCT patients, with backup on-call hematology/HCT physicians always available.

In the absence of undue or unmanageable toxicity, HCT patients remain “admitted” at the DH until neutrophil engraftment, with close follow-up arranged with their primary HCT physician following discharge. Patients requiring inpatient admission may return to the DH upon resolution of toxicity, be discharged home in case of engraftment, or for those requiring more prolonged toxicity management may discharge through a program known as “extended recovery after discharge” or ERAD, which allows for continued outpatient follow-up and medically-necessary hotel lodging for a finite period.

#### IEC therapy

For patients receiving IEC therapy, the process is fairly analogous to the autoHCT process, with a few key differences. Patients, either existing or referred for advanced therapies, are initially evaluated by a disease-specific, IEC-trained physician. All patients being considered for IEC therapy are presented to the Clinical Cellular and Immunotherapy Committee (CCIC), a multidisciplinary committee with IEC expertise and current clinical operations insight. This allows for assessment of appropriateness for commercial or investigational IEC therapies and outpatient versus inpatient administration, as well as contextualization within the broader resource environment. Dedicated IEC nurse coordinators for either commercial or investigational IEC therapies coordinate the workup, leukapheresis, and product shipment/receipt, before handing the patient over to the DH for outpatient LD chemotherapy and IEC administration, if the outpatient route is appropriate. APPs and HCT/IEC trained RNs provide ongoing care in the DH along with a rotating outpatient IEC physician who assesses patients daily.

### Roles and Responsibilities of HCT/IEC Team Members

#### HCT/IEC physicians

In the current practice of malignant hematology and HCT/IEC, there is a continually-shifting and challenging balance between maintenance of a longitudinal patient-physician relationship and the spatial-temporal limitations on individual physicians’ presence. In contrast to approaches that discretely separate HCT/IEC programs from non-cellular therapy malignant hematology, we see our “double physician appointment” model as allowing for continuity and oversight from a patient’s primary hematologist while simultaneously having the acute administration and management of IEC/HCT directed by discrete outpatient or inpatient teams led by hematologists on rotation (18). This, combined with regular committee meetings and touchpoints with leadership, strikes a nice balance between continuity and convenience.

Physicians within our hematology/HCT department are disease-focused in subsets of hematologic malignancies (eg. leukemia, lymphoma, or plasma cell dyscrasias), and have expertise in the ongoing management of patients within their disease focus using all treatment modalities, including HCT and IEC. Through having a global understanding of disease-specific treatment options (cellular and non-cellular therapies), the physicians are able to seamlessly direct patients to a specific treatment without requiring an extra outpatient consult. Additionally, they have expertise in the longitudinal management of complications and survivorship post HCT or IEC.

All physicians rotate on the inpatient and outpatient services. Most are disease-specific performing disease-specific HCT. However, the IEC inpatient and outpatients services are distinct, and given the overlapping management approaches and toxicities, all hematologists, irrespective of disease subspecialty, have the experience and ability to rotate on these services. Moreover, the disease-agnosticism of the IEC services and attendings also has allowed for inclusion of investigational IEC therapies in both solid and hematologic malignancies.

#### HCT nurse coordinators

The HCT nurse coordinators are disease specific. Those that specialize in multiple myeloma and lymphoma are generally involved in coordinating the workup and transplant for those intended for outpatient autoHCT. The details of their roles were described in the aforementioned paragraphs.

#### IEC nurse coordinators

The IEC nurse coordinator team plays a critical role in initiating, preparing and arranging essential care requirements prior to cellular therapy. Their roles consist of two phases with different sets of care coordination requirements: pre-leukapheresis phase and pre-lymphodepletion phase. Initial review and approval for CAR T cell therapy is obtained in CCIC meeting. During pre-leukapheresis phase, RN care coordinator initiates, submits and confirms completion of necessary paperwork, obtaining pathology results, ambulatory referral for CAR T cell work up in collaboration with treating team MD/APPs. They provide patient and caregiver education and are involved in the consenting process. They coordinate all the logistics ranging from registering patients onto the pharmaceutical company portal, generating purchase orders in collaboration with the pharmacy, working with financial department for authorization prior to leukapheresis, verification of counts requirement, washout windows, arranging vein assessments, line placement and apheresis. They collaborate with treating MDs once all screening reports become available and assist with ordering follow up testing if needed. They identify potential caregiver issues and collaborate with CSW and treating MD/APPs.

During Pre-LD phrase, RN care coordinators arrange for admission (as clinically indicated), local lodging (if needed) and collaborate with primary treating team (physician/APPs) for necessary outpatient oral medications. They also provide caregivers with education. They serve as a point of contact for patients and their caregivers prior to receiving lymphodepletion and cellular therapy. They also ensure and reinforce patients and caregivers with essential items and information such as the CAR T wallet card, driving restrictions, local stay and care giver requirements.

#### HCT APPs

The role of HCT APPs involve working with RN coordinators and physicians to coordinate sending transplant medications and educating patients/caregivers on medications via phone and providing written materials via email. Once patients are admitted to the DH to start their HCT, HCT APPs briefly orient them to the DH and the workflow, conditioning chemotherapy regimen, medications, their HCT course overview, what to expect while undergoing HCT in the outpatient setting and when to seek urgent medical attention. They reconcile medications diligently in collaboration with RNs and adjust accordingly. They review laboratory data and collaborate with the HCT rounding attending physician and pharmacists for any chemotherapy dose adjustments. They evaluate and manage HCT related toxicities in collaboration with the HCT rounding attending MD. They also utilize SOPs to guide their clinical practice. In the event of undue toxicity that cannot be managed adequately in the outpatient setting, or other medical or safety concern, they coordinate inpatient admission including communication with inpatient team, medication reconciliation, admission orders and writing the history and physical note.

#### APPs for commercial IEC product recipients

At the first encounter at our Day Hospital with patients who are to start LD and cellular therapy, Standard of Care (SOC) APPs orient them to DH, workflow/chemo/meds/CAR T overview/outpatient expectations/ETC or 911 precautions. In collaboration with IEC rounding attending MD, they evaluate and manage patients who are undergoing cellular therapy. They assess CAR T cell therapy related toxicities such as CRS or ICANS and arrange and coordinate for inpatient admission, when necessary. Their practice is guided by SOPs and collaborating physicians in caring for these patients with complex clinical needs.

They coordinate care to proactively address potential issues and barriers that can lead to a delay in care or treatment complications. They perform diligent medication reconciliation in collaboration with bedside RNs to ensure patients are on appropriate prophylaxis and supportive medications. They prescribe and refill essential medications needed for patients who remain in the outpatient setting for their CAR T therapy. They reinforce education on when to go to our Evaluation and Treatment Center for urgent management of CRS/ICANS or other situations that need urgent evaluation and care.

They coordinate admission including communication with inpatient team, medication reconciliation, admission orders and H&P whenever patients are required to be admitted to inpatient service for variety of reasons such as CRS or ICANS, neutropenic fever, symptom managements or other social factors (e.g. lack of consistent caregivers).

#### Research IEC APPs

The Research IEC APP is responsible for all patients who undergo cellular therapy under investigational and out-of-specification (OOS) commercial products. The role and responsibilities of research APPs is similar to those for the commercial-product APPs. In addition, the research APP collaborates with clinical research nurses on the specific clinical trial protocols and infusion RNs in the clinical research infusion center. The research APP must be knowledgeable, aware of restrictions and common practices associated with cellular therapies under investigational protocol. Both SOC and Research APPs work closely with inpatient APPs to ensure efficient and effective communication, patients hand-off and streamlined admissions and discharges. APPs in ambulatory settings are often required for management of co-morbidities and chronic diseases of patients undergoing CAR T cell therapies in the outpatient setting.

#### DH staff RNs

Day Hospital Staff RNs provide direct patient care such as nursing assessment, lab review, obtaining writing test and calculating immune cell encephalopathy (ICE) score, administering medications, HPC infusions, and blood product transfusions, and reinforcing detailed education, medication reviews and after care instructions to patients. They are guided by an extensive collection of SOPs with role-specific instructions.

### Opportunities for refinement and expansion

While we leverage our existing transplant infrastructure and expanding capacity to successfully and safely deliver cellular therapies in the ambulatory setting, all HCT/IEC centers are now faced with the challenge of increasing patient volumes, especially as the indications for CAR T expand. This underlying challenge contributes to a list of revolving obstacles, including physical space limitations, staffing shortages, shortages of chemotherapies and supportive medications, and leukapheresis slots availability. We and others have addressed some of the challenges through innovative strategies, such as staggering patient visits and rest days for stable patients out of the CRS window, but continued innovation will be needed.

We and others are incorporating “wearables” in an investigational capacity at this point. These ambulatory monitors are wireless, very small, and provide a link between the patient and monitoring center that can then triage patients toward appropriate provider based on vital sign instability or dynamics that could predict early toxicity. With refinement, these could potentially extend the radius from which patients can undergo transplant and CAR T, and allow patients to spend less time within clinic confines since there will be more longitudinal monitoring ongoing.

In addition to the HCT and CAR T/IEC programs, novel immunotherapies such as bispecific antibodies require intensive monitoring during the step-up dosing periods to manage acute inflammatory toxicities similar to CAR T. Ideally, these would be well-suited to conduct within the outpatient IEC program, although introduces volume challenges which need to be navigated.

## Actionable recommendations

1. Institutions should develop SOPs specifically to address outpatient HCT and IEC programs focusing on patient selection, caregiver education, staff/provider training, antibiotic prophylaxis, supportive care, toxicity management, indications for inpatient admission, and discharge pre-requisites.
2. There should be disease-specific and/or modality-specific consensus committees that review new patient cases, especially complex ones, that help determine the appropriateness of HCT or IEC and provide context relating to the available resources.
3. Based on our experience, we support models in which disease-specific physicians (eg. Multiple myeloma specialists) also have training and experience in HCT and IEC administration/management as relevant to their disease focus. This allows for longitudinal, uninterrupted care of one’s own patients regardless of treatment modality as well as ability to attend on HCT/IEC services. This model may function in some institutions better than others.
4. Social workers should be involved early in the patient selection process because they can identify caregiver, lodging, and socio-economic barriers that may be easier to overcome in the earlier stages with advanced planning.
5. Dedicated HCT and IEC coordinators quarterback the entire process from consultation to survivorship, and are integral to cohesiveness and continuity of the process.
6. Quality reviews should be held regularly to review early mortality or excessive morbidity that could reflect systematic issues requiring improvement.

## Special considerations for resource-constrained settings

While our institution and others are fortunate to practice in resource-rich environments, we acknowledge that many patients with hematologic malignancies receive care in low and middle-income countries (LMICs). In many of these LMICs, transplant programs have had to adopt outpatient transplant models out of necessity rather than out of convenience in order to take advantage of the cost savings and resource preservation that these models afford (19). As such, some of the pioneering developments of outpatient HCT occurred within LMICs (20). One such program is that of Mexico’s Clínica Ruiz which initiated an HCT program in 1993, starting with AutoHCT for MM, then expanding to AutoHCT for autoimmune disease, and then allogeneic HCT for hematologic malignancies thereafter (21, 22). They have published extensively on the feasibility and success of their outpatient HCT program. Many of the prerequisites overlap with those highlighted above including rigorous patient selection, caregiver availability and education, clinic or “day hospital” open every day of the week, 24-hour triage line and physician availability, and an available hospital should inpatient care be required. These basic requirements are within reach of programs within resource-constrained settings, and highlight the pivotal role of personnel and training. As demonstrated, elements that may seem integral to programs within resource-rich settings, may actually not impede the development of an outpatient HCT program. For example, many programs within LMICs do not cryopreserve the apheresis product, which in their context is unnecessary, and allows them to direct resources elsewhere (6, 23, 24). Similarly, in the allogeneic HCT setting, given comparably favorable efficacy and safety data, they favor haploidentical HCT over umbilical cord blood HCT, due to the cost and quality challenges of local cord blood banking, and over matched unrelated donor HCT given the expense and difficulty of seeking and transporting matched unrelated grafts to LMICs. Additionally, they lack advanced practice providers and therefore have to delegate duties among registered nurses and physicians, with an emphasis on training and collaboration. Despite lack of some of these conveniences, they nevertheless have proven the ability to successfully conduct HCT in the outpatient setting.

The next frontier for these outpatient HCT programs in LMICs will be the translation of their successful platforms toward adopting IEC programs in the outpatient setting. At this point it remains unclear how the resource intensive elements of CAR T/IEC therapy might be pared down in order to accommodate the abilities of LMIC programs. For example, CAR T therapies, at this point, require cryopreservation and shipment to a pharmaceutical partner for manufacturing; however, on-site CAR T manufacturing or some other innovation could potentially mitigate these barriers. Additionally, the management of CAR T toxicities can often prove costly and require prolonged inpatient hospitalization and expensive therapies such as tocilizumab and other biologic agents. Protocols that incorporate inexpensive prophylactic measures such as a prophylactic dexamethasone for CRS prevention could reduce expenditures and make CAR T/IEC therapy more tangible in resource-constrained environments (25).

## Conclusions

As many institutions have demonstrated, outpatient programs for AutoHCT and IEC are feasible, safe, and cost-effective. The consistent pillars of success include highly-trained providers and staff who have expertise in their specific roles as well as an understanding of the responsibilities of all involved, SOPs that clearly delineate candidacy for the outpatient programs, proactive measures to minimize risks of infection and other toxicities, and an infrastructure that supports the patient and caregiver through ongoing education, communication, and connection.

The current outpatient programs serve as templates from which other institutions can build theirs. Nuances related to locality and population will contribute to the details and differences among individual programs, however the main goals of optimizing outcomes for patients and systems remain the same. Those that aim to develop programs in resource-constrained environments can model the highly-published LMIC programs which have been successful at dividing necessities from conveniences in order to maximize resource optimization and minimize expenditure without compromising care. The initial success that has been demonstrated should encourage the expansion of outpatient HCT and IEC programs to improve the accessibility and sustainability of these highly effective therapies.

## Author contributions

SG: Conceptualization, Writing – original draft, Writing – review & editing. MS-R: Writing – original draft, Writing – review & editing. JK: Writing – original draft, Writing – review & editing. JR: Writing – original draft, Writing – review & editing. MR: Conceptualization, Writing – original draft, Writing – review & editing.
